# Reported Estimates of Adverse Pregnancy Outcomes among Women with and without Syphilis: A Systematic Review and Meta-Analysis

**DOI:** 10.1371/journal.pone.0102203

**Published:** 2014-07-15

**Authors:** Jiabi Qin, Tubao Yang, Shuiyuan Xiao, Hongzhuan Tan, Tiejian Feng, Hanlin Fu

**Affiliations:** 1 Department of Epidemiology and Health Statistics, School of Public Health, Central South University, Hunan, China; 2 Department of Social Medicine, School of Public Health, Central South University, Hunan, China; 3 Department of Dermatology and Venereal Disease, Shenzhen Center for Chronic Disease Control and Prevention, Shenzhen, China; Seattle Childrens Hospital, United States of America

## Abstract

**Background:**

To estimate probability of adverse pregnancy outcomes (APOs) among women with and without syphilis through a systematic review of published literatures.

**Methodology/Principal Findings:**

Chinese and English literatures were searched for studies assessing pregnancy outcomes in the presence of maternal syphilis through August 2013. The prevalence estimates were summarized and analyzed by meta-analysis. Fifty-four literatures involving 11398 syphilitic women and 43342 non-syphilitic women were included from 4187 records initially found. *Among untreated mothers with syphilis*, pooled estimates were 76.8% for all APOs, 36.0% for congenital syphilis, 23.2% for preterm, 23.4% for low birth weight, 26.4% for stillbirth or fetal loss, 14.9% for miscarriage and 16.2% for neonatal deaths. *Among syphilitic mother receiving treatment only in the late trimester (>28 weeks)*, pooled estimates were 64.4% for APOs, 40.6% for congenital syphilis, 17.6% for preterm, 12.4% for low birth weight, and 21.3% for stillbirth or fetal loss. *Among syphilitic mothers with high titers (≥1∶8)*, pooled estimates were 42.8% for all APOs, 25.8% for congenital syphilis, 15.1% for preterm, 9.4% for low birth weight, 14.6% for stillbirth or fetal loss and 16.0% for neonatal deaths. A*mong non-syphilitic mothers*, the pooled estimates were 13.7% for all APOs, 7.2% for preterm birth, 4.5% for low birth weight, 3.7% for stillbirth or fetal loss, 2.3% for miscarriage and 2.0% for neonatal death. Begg's rank correlation test indicated little evidence of publication bias (*P*>0.10). Substantial heterogeneity was found across studies in the estimates of all adverse outcomes for both women with syphilis (*I* ^2^ = 93.9%; *P*<0.0001) and women without syphilis (*I* ^2^ = 94.8%; *P*<0.0001).

**Conclusions/Significance:**

Syphilis continues to be an important cause of substantial perinatal morbidity and mortality, which reminds that policy-makers charged with resource allocation that the elimination of mother-to-child transmission of syphilis is a public health priority.

## Introduction

Mother-to-child transmission (MTCT) of syphilis has been documented since the 15th century, yet, today, continues to cause substantial perinatal morbidity and mortality, even in developed countries, where antenatal health services are strong [1]–[2]. Prenatal screening coupled with appropriate, prompt penicillin treatment in prevention of MTCT of syphilis is feasible, inexpensive, and cost-effective, even in settings where the burden of syphilis among pregnant women is moderate or low [3]–[5]. Yet, despite the tools being available for over 60 y, MTCT of syphilis persists as a public health problem in many rural, urban, and suburban communities [9]. In 2007, the World Health Organization (WHO) launched its initiative for Global Elimination of Congenital Syphilis (CS) as millennium development goals [6]. In response to the call of the WHO, in June 2010, the China's Ministry of Health (MOH) officially launched the first national program specially and directly aimed at controlling syphilis and blocking MTCT of syphilis: the National Program for Prevention and Control of Syphilis in China (2010–2020) [7]. In order to effectively eliminate of MTCT of syphilis and to guide policy and advocacy efforts, global data on the burden of syphilis in pregnancy and associated adverse pregnancy outcomes (APOs) are needed.

Currently, although some estimates for burden of syphilis in pregnancy and associated APOs were available, the global incidence of adverse birth outcomes among syphilitic women remains enough unclear. Over the years, some researchers have been trying to estimate the incidence of APOs resulting from maternal syphilis. For example, the latest meta-analysis by Gomez et al. [8] that is currently the only systematic review assessing this question indicated that approximately 52% of pregnancies in mothers with untreated or inadequately treated syphilis result in some APOs, with estimated proportions: early fetal loss or stillbirth (21%), neonatal death (9%), low birth weight or premature birth (6%), and infection in a live-born infant (15%), and among untreated pregnant women with syphilis, fetal loss and stillbirth were 21% more frequent, neonatal deaths were 9.3% more frequent and prematurity or low birth weight were 5.8% more frequent than among women without syphilis. However, this review didn't assess APOs under the background of different baseline titers and treatment time for maternal syphilis, and didn't include Chinese literatures. In China, there are large study reports to assess adverse outcomes among women with syphilis, while China's literatures are mainly local or single medical institution reports and only include the small sample size of study population, which causes that the findings are not comprehensive and meaningful representation of poor. Overall, most of previous estimates of APOs in women with syphilis have been based on point estimates from single studies.

Today, the full extent of MTCT of syphilis is difficult to measure because there is no definitive test for MTCT transmission; diagnosis based on clinical history and serologic testing in mothers and infants is often unavailable. Additionally, the countries where CS continues to be most problematic often lack even basic testing capacity, let alone more sophisticated laboratory techniques for diagnosis and staging syphilis during pregnancy. However, the large body of literatures from China, where syphilis testing is routinely done during pregnancy, can help address this issue. In order to support the global initiative for elimination of MTCT of syphilis, we conducted a systematic review and meta-analysis with the following objectives: (1) to estimate the APOs among syphilitic women according to baseline titers, treatment or not during pregnancy and gestational age at treatment; (2) to estimate APOs among women without syphilis; and (3) to provide scientific evidence for the prevention of pregnancy loss attributable to MTCT of syphilis.

## Methods

### Search strategy

PubMed, Cochrane Libraries, China Biology Medicine disc (CBMdisc), Chinese Scientific Journals Fulltext Database (CQVIP), China National Knowledge Infrastructure (CNKI) and Wanfang Data were searched through August 2013 with no restrictions to identify published peer-reviewed research articles assessing pregnancy outcomes in the presence of maternal syphilis by the following search terms: syphilis, pregnancy, adverse birth or pregnancy outcomes, congenital syphilis, preterm, low birth weight, stillbirth, fetal loss or death, abortion or miscarriage, neonatal death, and perinatal death or morbidity or mortality. We also performed a manual search on the reference lists of published articles. The grey literature and conference abstracts were not searched. This review was conducted and reported according to MOOSE guidelines and PRISMA requirements [10]–[11].

### Selection criteria

For those studies that not only reported the incidence of birth outcomes among syphilis-infected women, but also reported the incidence among non-syphilitic women, we will meanwhile estimate the range of possible birth outcomes among non-syphilitic women. Our APOs of interest were CS, preterm, low birth weight, stillbirth or early fetal loss, miscarriage and neonatal death. Studies were considered eligible for inclusion in this systematic review if they met the following criteria: (1) studies published in Chinese or English language; (2) studies described pregnancy outcomes among women presumed to have syphilis(i.e. women who were seroreactive for T. pallidum infection, irrespective of the test used); (3) study populations excluded HIV-positive women; (4) sample size for cases was more than 30 syphilitic patients; and (5) the incidences of APOs were reported(or data to calculate them). We excluded review papers, non peer–reviewed local/government reports, conference abstract and presentation in this study. If the same study data were published in both English and Chinese sources, the articles published in Chinese language were excluded from the review. We considered a broad range of study designs, including clinical trials, observational studies, and case series. We also assessed potential studies to ensure that there was no duplication of case series.

### Data extraction

Two independent reviewers (JBQ and HLF) assessed eligibility criteria and extracted data, and any disagreements were resolved by discussion. We extracted the following information from all eligible studies: first author and published year; geographical location; study design; study period; syphilis prevalence among mothers; subgroup variables (infection status of syphilis, treatment or not for maternal syphilis, gestational age at treatment, and baseline titers of nontreponemal antibodies); sample size for cases and controls; reported adverse outcomes. Because variations in the definition of APOs exist across countries and cultures, it is extremely difficult to define uniform standards. The early literatures did not always define birth outcomes and in such cases we relied on the outcome terminology in the original papers.

### Statistical analysis

We calculated the combined incidence and the corresponding 95% confidence intervals (CI) for all APOs in women with syphilis and women without syphilis. We then also calculated the summary incidence and the corresponding 95%CI for the following selected pregnancy loss. The subgroup analysis for all APOs and specific APOs was performed based on whether women were infected with syphilis, whether syphilitic women were treated during pregnancy (i.e. syphilitic women receiving at least one injection of 2.4 million units of penicillin before delivery), gestational week at treatment (i.e. <12 or 12 to 28 or ≥28 weeks), and maternal baseline titers (i.e. ≥1∶8 *or* <1∶8) to explore the sources of heterogeneity.

The combined incidence and the corresponding 95% CI were calculated using either fixed-effects models or, in the presence of heterogeneity, random-effects models. Heterogeneity tests were performed using the Cochran Q-test (p<0.10 represents statistically significant heterogeneity) and I^2^ statistic. Begg's rank correlation test was used to assess publication bias (p<0.10 represents statistical significance). The chi-square test was used to analyze the difference between subgroups (p<0.05 represents statistical significance). The comparison between subgroups was performed using SAS version 9.1, and other data were prepared and analyzed using R software version 3.0.

## Results

### Study characteristics

**Figure 1:**
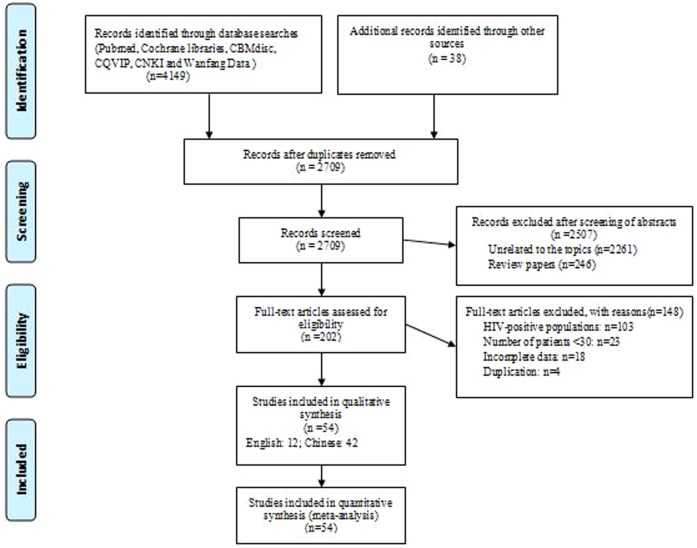
Flow chart showing the meta-analysis studies selection. n, the number of prevalence estimates included in meta-analysis.

Our initial search criteria identified 4149 articles from six electronic databases and 38 additional articles were identified through reference lists from identified articles. Of these, the majority were excluded after the first screening based on abstracts or titles, mainly because they were review papers, and unrelated to the topics or duplicated titles from different databases (Figure 1). Finally, fifty-four studies [12]–[19], [21]–[24], [32]–[73] were considered eligible in qualitative synthesis. The characteristics of included studies involving 11398 women with syphilis and 43342 women without syphilis and published between 1917 and 2013 were summarized in Table 1. Forty-five studies [22]–[24], [32]–[73] were conducted in China, one [12] in UK, three in USA [13]-[14], [17], one [15] in Zambia, one [16] in Malawi, one [18] in Kenya, one [19] in Tanzania, and one [21] in Russia. All articles belonged to observational studies including retrospective cohort studies, retrospective cases analysis, prospective cohort studies, and prospective surveillance. Twelve studies (21.8%) presented the findings of observational studies that included a “control” arm assessing APOs among women without syphilis. Syphilis prevalence among mothers was reported from 22.1 to 765.7 cases per 10000 pregnant women. Forty-six studies reported on clinical evidence of CS in children. Thirty-four studies reported on preterm birth and fourteen studies reported on low birth weight. Forty-one studies reported on stillbirth or early fetal loss and fifteen studies reported on miscarriage. Twenty studies reported on neonatal death.

**Table 1:** Characteristics of studies included in a systematic review and meta-analysis to determine the frequency of adverse pregnancy outcomes (APOs) among women with syphilis and women without syphilis.

Study	Location	Study design	Period	Syphilis prevalence among mothers (1/10000)	Sample size	Subgroup variables	Reported adverse pregnancy outcomes (APOs)
Harman/1917 [12]	United kingdom	retrospective cohort	1917	360.0	Syphilitic mothers: 1001	mothers with syphilis or without syphilis	Congenital syphilis, and stillbirth and fetal loss, and all APOs
					Non-syphilitic mothers: 826		
Wammock/1950 [13]	United states of America	retrospective cohort	1045–1948	150.0	Syphilitic mothers: 61	mothers with syphilis or without syphilis	Congenital syphilis, preterm birth or low birth weight, stillbirth or fetal loss, and neonatal death, and all APOs
					Non-syphilitic mothers: 5596		
Ingraham/1950 [14]	United states of America	Prospective cohort	1940–1949	150.0	Syphilitic mothers: 220	mothers with syphilis or without syphilis	Congenital syphilis, Stillbirth or fetal loss, neonatal death, preterm birth or low birth weight, and all APOs
					Non-syphilitic mothers: 10323		
Hira/1990 [15]	Zambia	Prospective surveillance	1985–1987	Unknown	Syphilitic mothers: 230	mothers with syphilis or without syphilis	Congenital syphilis, preterm birth, low birth weight, stillbirth or fetal loss, miscarriage, and all APOs
					Non-syphilitic mothers: 2647		
McDermott/1993 [16]	Malawi	retrospective cohort	1987–1990	362.0	Syphilitic mothers: 130	mothers with syphilis or without syphilis	Stillbirth or fetal loss, and neonatal death, and all APOs
					Non-syphilitic mothers: 3591		
Barbara/1995 [17]	United states of America	Retrospective analysis	1991–1992	Unknown	Syphilitic mothers: 253	mothers with syphilis or without syphilis	Congenital syphilis, stillbirth or fetal loss, and neonatal death
					Non-syphilitic mothers: 7929		
Temmerman/2000 [18]	Kenya	Prospective cohort	1997–1998	238.4	Syphilitic mothers: 275	mothers with syphilis or without syphilis	Low birth weight, and stillbirth or fetal loss
					Non-syphilitic mothers: 275		
Deborah WJ/2002 [19]	Tanzania	Prospective cohort	1997–1999	765.7	Syphilitic mothers: 382	gestational week at treatment; mothers with syphilis or without syphilis; baseline titers of nontreponemal antibodies	Preterm birth, low birth weight, stillbirth or fetal loss, and all APOs
					Non-syphilitic mothers: 950		
Tikhonova/2003 [21]	Russia	retrospective cohort	1995–1999	Unknown	Syphilitic mothers: 628	treatment or not	Stillbirth or fetal loss
Liu JB/2010 [22]	Shenzhen,China	Prospective cohort	2002–2007	43.4	Syphilitic mothers: 554	baseline titers of nontreponemal antibodies	Congenital syphilis
Zhu LP/2010 [23]	Shanghai, China	Prospective cohort	2002–2006	27.5	Syphilitic mothers: 1471	treatment or not;baseline titers of nontreponemal antibodies;gestational week at treatment	Congenital syphilis
Qin JB/2013 [24]	Shenzhen, China	Prospective cohort	2007–2012	30.0	Syphilitic mothers: 360	treatment or not;baseline titers of nontreponemal antibodies;gestational week at treatment	Congenital syphilis
Lv J/2001 [32]	Guangzhou	Retrospective analysis	1994–2000	Unknown	Syphilitic mothers: 64	treatment or not	Congenital syphilis, preterm birth, stillbirth or fetal loss, and all APOs
Xu Y/2001 [33]	Haikou	Retrospective analysis	1995–2001	62.2	Syphilitic mothers: 48	treatment or not	Congenital syphilis, preterm birth, stillbirth or fetal loss, miscarriage, neonatal death, and all APOs
Lin XH/2002 [34]	Guangzhou	Retrospective analysis	1998–2000	74.0	Syphilitic mothers: 41	treatment or not; mothers with syphilis or without syphilis	Congenital syphilis, preterm birth, stillbirth or fetal loss, neonatal death, and all APOs
					Non-syphilitic mothers: 5532		
Fang SN/2003 [35]	Shenzhen	Retrospective analysis	1997–2002	Unknown	Syphilitic mothers: 42	treatment or not	Congenital syphilis, preterm birth, stillbirth or fetal loss, miscarriage, and all APOs
Wang HB/2003 [36]	Shanghai	Retrospective analysis	1998–2002	51.2	Syphilitic mothers: 21	treatment or not	Congenital syphilis
Kuang YB/2004 [37]	Guangzhou	Prospective cohort	2001–2003	135.4	Syphilitic mothers: 73	mothers with syphilis or without syphilis	Preterm birth, stillbirth or fetal loss, and all APOs
					Non-syphilitic mothers: 5317		
Xu YX/2004 [38]	Shenzhen	Prospective cohort	2002–2003	Unknown	Syphilitic mothers: 54	treatment or not	Congenital syphilis, stillbirth or fetal loss, miscarriage, neonatal death, and all APOs
Zhang XM/2004 [39]	Fuzhou	Prospective cohort	1996–2001	69.7	Syphilitic mothers: 192	treatment or not;baseline titers of nontreponemal antibodies;gestational week at treatment	Congenital syphilis, preterm birth, low birth weight, stillbirth or fetal loss, miscarriage, neonatal death, and all APOs
Li Q/2005 [40]	Dongguan	Retrospective analysis	2003–2004	Unknown	Syphilitic mothers: 46	mothers with syphilis or without syphilis	Congenital syphilis, preterm birth, low birth weight, stillbirth or fetal loss, neonatal death, and all APOs
					Non-syphilitic mothers: 356		
Zhou H/2006 [41]	Shenzhen	Prospective cohort	2002–2004	42.9	Syphilitic mothers: 371	baseline titers of nontreponemal antibodies;gestational week at treatment	Congenital syphilis
Gao H/2006 [42]	Zhanjiang	Retrospective analysis	2002–2005	Unknown	Syphilitic mothers: 97	gestational week at treatment	Congenital syphilis, preterm birth, low birth weight, stillbirth or fetal loss, and all APOs
Wang CX/2006 [43]	Guangzhou	Retrospective analysis	1997–2005	Unknown	Syphilitic mothers: 48	treatment or not	Congenital syphilis, preterm birth, stillbirth or fetal loss, miscarriage, and all APOs
Xuan QS/2006 [44]	Guangzhou	prospective surveillance	1995–2003	Unknown	Syphilitic mothers: 286	gestational week at treatment	Congenital syphilis, preterm birth, low birth weight, stillbirth or fetal loss, miscarriage, neonatal death, and all APOs
Zheng RQ/2006 [45]	Shenzhen	Retrospective analysis	2000–2005	87.0	Syphilitic mothers: 48	treatment or not	Congenital syphilis, preterm birth, stillbirth or fetal loss, miscarriage, neonatal death, and all APOs
Wang X/2007 [46]	Shantou	Retrospective analysis	Unknown	Unknown	Syphilitic mothers: 68	treatment or not	Congenital syphilis, preterm birth, stillbirth or fetal loss, and all APOs
Sun LL/2008 [47]	Shaoguan	Retrospective analysis	2000–2006	91.7	Syphilitic mothers: 62	treatment or not	Congenital syphilis, preterm birth, miscarriage, neonatal death, and all APOs
Gao JM/2009 [48]	Nanchang	prospective surveillance	2003–2007	Unknown	Syphilitic mothers: 82	no	Congenital syphilis, preterm birth, low birth weight, stillbirth or fetal loss, neonatal death, and all APOs
Huang ZM/2009 [49]	Shenzhen	Retrospective analysis	2005–2007	Unknown	Syphilitic mothers: 452	treatment or not;baseline titers of nontreponemal antibodies	Congenital syphilis
Li L/2009 [50]	Beijing	Retrospective analysis	2006–2007	Unknown	Syphilitic mothers: 121	treatment or not;gestational week at treatment	Congenital syphilis, preterm birth, stillbirth or fetal loss, and all APOs
Wu FY/2009 [51]	Zhejiang	Retrospective analysis	2006–2008	97.7	Syphilitic mothers: 47	treatment or not	Congenital syphilis, preterm birth, low birth weight, stillbirth or fetal loss, neonatal death, and all APOs
Zhou GJ/2009 [52]	Hefei	Retrospective analysis	2003–2006	110.3	Syphilitic mothers: 53	treatment or not	Congenital syphilis, preterm birth, low birth weight, stillbirth or fetal loss, neonatal death, and all APOs
Chen JH/2010 [53]	Liuyang	prospective surveillance	2008–2009	77.6	Syphilitic mothers: 61	gestational week at treatment	Congenital syphilis, preterm birth, stillbirth or fetal loss, miscarriage, and all APOs
Li TH/2010 [54]	Huhehaote	prospective surveillance	2006–2009	Unknown	Syphilitic mothers: 168	treatment or not;gestational week at treatment	Congenital syphilis, preterm birth, stillbirth or fetal loss, and all APOs
Shuang JY/2010 [55]	Taiyuan	Retrospective analysis	2006–2010	Unknown	Syphilitic mothers: 48	treatment or not;baseline titers of nontreponemal antibodies	Congenital syphilis, and all APOs
Ye GR/2010 [56]	Panzhihua	Prospective cohort	2008–2010	Unknown	Syphilitic mothers: 80	gestational week at treatment	Preterm birth, low birth weight, stillbirth or fetal loss, and all APOs
Dai Y/2011 [57]	Yangzhou	Retrospective analysis	2006–2010	60.3	Syphilitic mothers: 136	gestational week at treatment	Congenital syphilis, and all APOs
Li Z/2011 [58]	Shenzhen	Prospective cohort	2002–2010	26.2	Syphilitic mothers: 427	treatment or not	Congenital syphilis
Luo ZZ/2011 [59]	Shenzhen	Prospective cohort	2007–2010	23.7	Syphilitic mothers: 227	gestational week at treatment; baseline titers of nontreponemal antibodies	All APOs
Wang WL/2011 [60]	Zhejiang	Retrospective analysis	2006–2009	Unknown	Syphilitic mothers: 52	treatment or not; gestational week at treatment	Congenital syphilis, preterm birth, stillbirth or fetal loss, and all APOs
Yuan XQ/2011 [61]	Chengdu	Retrospective analysis	2010–2011	Unknown	Syphilitic mothers: 52	gestational week at treatment	Congenital syphilis, preterm birth, stillbirth or fetal loss, and all APOs
Cao DH/2012 [62]	Zhongshan	Retrospective analysis	2005–2010	Unknown	Syphilitic mothers: 41	treatment or not	Congenital syphilis, preterm birth, low birth weight, stillbirth or fetal loss, miscarriage, neonatal death, and all APOs
Chen GJ/2012 [63]	Shenzhen	Prospective cohort	2004–2009	48.7	Syphilitic mothers: 330	treatment or not	Congenital syphilis, preterm birth, stillbirth or fetal loss, miscarriage, and all APOs
Deng JF/2012 [64]	Shenzhen	Retrospective analysis	2009–2011	Unknown	Syphilitic mothers: 58	treatment or not	Congenital syphilis, preterm birth, and stillbirth or fetal loss, miscarriage, neonatal death, and all APOs
Li HS/2012 [65]	Changchun	Retrospective analysis	2006–2011	22.1	Syphilitic mothers: 33	treatment or not	Preterm birth, stillbirth or fetal loss, neonatal death, and all APOs
Li Z/2012 [66]	Chongzuo	Retrospective analysis	2004–2011	Unknown	Syphilitic mothers: 86	gestational week at treatment	Congenital syphilis, and all APOs
Pan P/2012 [67]	Shenzhen	Prospective cohort	2005	Unknown	Syphilitic mothers: 584	no	Congenital syphilis, and all APOs
Xu ZY/2012 [68]	Shenzhen	Prospective cohort	2005–2010	Unknown	Syphilitic mothers: 772	gestational week at treatment; baseline titers of nontreponemal antibodies	Congenital syphilis, preterm birth, low birth weight, stillbirth or fetal loss, neonatal death, and all APOs
Cui L/2013 [69]	Xinxiang	Retrospective analysis	2007–2012	Unknown	Syphilitic mothers: 80	treatment or not	Congenital syphilis, preterm birth, stillbirth or fetal loss, and all APOs
Shi J/2013 [70]	Guangzhou	Retrospective analysis	2006–2011	Unknown	Syphilitic mothers: 85	gestational week at treatment	Congenital syphilis, preterm birth, stillbirth or fetal loss, miscarriage, neonatal death, and all APOs
Wei HP/2013 [71]	Beihai	Prospective cohort	2010–2012	Unknown	Syphilitic mothers: 89	gestational week at treatment; baseline titers of nontreponemal antibodies	Congenital syphilis, preterm birth, stillbirth or fetal loss, miscarriage, and all APOs
Wu FY/2013 [72]	Qujing	prospective surveillance	2009–2011	Unknown	Syphilitic mothers: 56	treatment or not	Congenital syphilis, preterm birth, stillbirth or fetal loss, and all APOs
Xu ZY/2013 [73]	Zhejiang	prospective surveillance	2009–2011	Unknown	Syphilitic mothers: 52	treatment or not	Congenital syphilis, preterm birth, low birth weight, neonatal death, and all APOs

### All APOs among women with and without syphilis

The reported proportion range of all APOs in the original studies is from 12.3% to 95.1% with a median of 49.2% among women with syphilis and from 9.3% to 20.8% with a median of 12.5% among women without syphilis (Table 2). The pooled estimates of all APOs were 47.7% (95%CI: 41.6–54.0) among syphilis-infected women and 13.7% (95%CI: 12.0–15.6) among women without syphilis (Table 2), for an absolute difference of 34.0% (χ^2^ = 3616.129, *P* = 0.000) (Table 3). Begg's rank correlation test indicated little evidence of publication bias (*P* = 0.171 to 0.397) for summary estimates of all APOs among women with and without syphilis (Table 2). Substantial heterogeneity was found across studies in the estimates of all APOs for both women with syphilis (*I* ^2^ = 93.9%; *P*<0.0001) and women without syphilis (*I* ^2^ = 94.8%; *P*<0.0001).

**Table 2:** Summary estimates of the proportion (%) of adverse pregnancy outcomes (APOs) among women with syphilis and women without syphilis.

	Reported proportion in the original studies		APOs	n	No. of included studies	Summary estimates (95%CI)#	Heterogeneity	Bias assessment
	Range	M (IQR)						
Women with syphilis								
All APOs	12.3%–95.1%	49.2% (58.0%–37.0%)	2495	5237	41	47.7% (95%CI: 41.6%–54.0%)	I^2^ = 93.9%, *P*<0.0001	*P* = 0.397
Congenital syphilis	0.6%–79.3%	20.6% (38.2%–10.3%)	1680	9430	46	20.6% (95%CI: 16.4%–25.6%)	I^2^ = 95.3%, *P*<0.0001	*P* = 0.403
Preterm birth	0.9%–39.4%	15.3% (19.2%–11.0%)	451	4089	34	14.1% (95%CI: 11.4%–17.3%)	I^2^ = 81.6%, *P*<0.0001	*P* = 0.272
Low birth weight	3.6%–29.3%	17.0% (21.3%–5.8%)	288	2593	14	13.2% (95%CI: 9.2%–18.5%)	I^2^ = 89.3%, *P*<0.0001	*P* = 0.133
Miscarriage	3.1%–14.8%	5.6% (10.3%–3.7%)	109	1674	15	6.6% (95%CI: 4.7%–9.3%))	I^2^ = 66.0%, *P = 0.0002*	*P* = 0.194
Stillbirth or fetal loss	2.1%–43.8%	12.1% (21.6%–7.3%)	802	6558	41	12.5% (95%CI: 10.0%–15.5%)	I^2^ = 88.9%, *P*<0.0001	*P* = 0.391
Neonatal death	1.0%–33.3%	5.5% (13.4%–2.6%)	140	2413	20	6.6% (95%CI: 4.1%–10.4%)	I^2^ = 84.2%, *P*<0.0001	*P* = 0.107
Women without syphilis								
All APOs	9.3%–20.8%	12.5% (17.7%–10.9%)	4640	34546	9	13.7% (95%CI: 12.0%–15.6%)	I^2^ = 94.8%, *P*<0.0001	*P* = 0.171
Preterm birth	3.0%–11.8%	7.6% (9.7%–4.1%)	1201	15011	5	7.2% (95%CI: 5.6%–9.3%)	I^2^ = 93.6%, *P*<0.0001	*P* = 0.102
Low birth weight	1.8%–9.9%	5.1% (9.5%–1.9%)	166	4313	4	4.5% (95%CI: 2.0%–10.0%)	I^2^ = 95.9%, *P*<0.0001	*P* = 0.142
Miscarriage			62	2647	1	2.3% (95%CI: 1.8%–3.0%)		
Stillbirth or fetal loss	1.1%–9.4%	3.6% (6.4%–1.7%)	1536	42726	11	3.7% (95%CI: 2.6%–5.1%)	I^2^ = 97.3%, *P*<0.0001	*P* = 0.230
Neonatal death	0.8%–4.1%	2.2% (3.6%–0.8%)	581	27094	5	2.0% (95%CI: 1.2%–3.3%)	I^2^ = 96.9%, *P*<0.0001	*P* = 0.181

M = Median; IQR = Inter-quartile range; APOs =  adverse pregnancy outcomes; CI = Confidence interval.

# Summary estimates and their corresponding 95% CI were calculated using either fixed-effects models or, in the presence of heterogeneity, random-effects models.

**Table 3:** Comparison for summary estimates of the proportion (%) of adverse pregnancy outcomes (APOs) among different subgroups.

Subgroup	All APOs		Congenital syphilis		Preterm birth		Low birth weight	
	absolute differences	chi-square test	absolute differences	chi-square test	absolute differences	chi-square test	absolute differences	chi-square test
Women with syphilis *vs* women without syphilis	34.0%	?^2^ = 3616.129, *P* = 0.000			6.9%	*χ* ^2^ = 37.312, *P* = 0.000	8.7%	*χ* ^2^ = 138.897, *P* = 0.000
Untreated women with syphilis *vs* women without syphilis	63.1%	?^2^ = 3947.821, *P* = 0.000			16.0%	?^2^ = 139.350, *P* = 0.000	18.9%	?^2^ = 110.776, *P* = 0.000
Treatment in the third trimester *vs* women without syphilis	50.7%	?^2^ = 727.296, *P* = 0.000			10.4%	?^2^ = 24.696, *P* = 0.000	7.9%	?^2^ = 28.440, *P* = 0.000
High titers (≥1∶8) *vs* women without syphilis	29.1%	?^2^ = 209.950, *P* = 0.000			7.9%	?^2^ = 18.044, *P* = 0.000	4.9%	?^2^ = 22.790, *P* = 0.000
Untreated women with syphilis *vs* Treated women with syphilis	52.6%	?^2^ = 1059.165, *P* = 0.000	22.0%	?^2^ = 290.433; *P* = 0.000	13.3%	*χ* ^2^ = 66.595, *P* = 0.000	17.2%	*χ* ^2^ = 53.604,*P* = 0.000
Treatment in the third trimester *vs* treatment in the first trimester	51.1%	?^2^ = 126.190, *P* = 0.000	31.0%	?^2^ = 69.475, *P* = 0.000	10.8%	*χ* ^2^ = 8.885, *P* = 0.003	2.4%	*χ* ^2^ = 0.020, *P* = 0.889
High titers (≥1∶8) *vs* Low titers (<1∶8)	31.8%	?^2^ = 174.840, *P* = 0.000	21.6%	?^2^ = 283.664, *P* = 0.000	12.2%	*χ* ^2^ = 55.631, *P* = 0.000	5.5%	*χ* ^2^ = 13.853, *P* = 0.000
Gestational week at treatment for women with syphilis		?^2^ *_trend_* = 140.168, *P* = 0.000		?^2^ *_trend_* = 95.126, *P* = 0.000		*χ^2^_trend_* = 12.509, *P* = 0.000		*χ^2^_trend_* = 3.402, *P* = 0.065

### Selected APOs among women with and without syphilis

The pooled estimates were 20.6% (95%CI: 16.4–25.6) for CS, 14.1% (95%CI: 11.4–17.3) for preterm, 13.2% (95%CI: 9.2–18.5) for low birth weight, 12.5% (95%CI: 10.0–15.5) for stillbirth or early fetal loss, 6.6% (95%CI: 4.7–9.3) for miscarriage and 6.6% (95%CI: 4.1–10.4) for neonatal deaths among mothers with syphilis (Table 2). For mothers without syphilis, the pooled estimates were 7.2% (95%CI: 5.6–9.3) for preterm, 4.5% (95%CI:2.0–10.0) for low birth weight, 3.7% (95%CI: 2.6–5.1) for stillbirth or early fetal loss, 2.3% (95%CI: 1.8–3.0) for miscarriage and 2.0% (95%CI: 1.2–3.3) for neonatal deaths (Table 2). The absolute differences between syphilitic mothers and non-syphilitic mothers for preterm birth (*χ* ^2^ = 37.312, *P* = 0.000), low birth weight (*χ* ^2^ = 138.897, *P* = 0.000), stillbirth or fetal loss (*χ* ^2^ = 937.960, *P* = 0.000), miscarriage (*χ* ^2^ = 46.895, *P* = 0.000), and neonatal death (*χ* ^2^ = 124.340, *P* = 0.000) were 6.9%, 8.7%, 8.8%, 4.3%, and 4.6%, respectively (Table 3 and Table 4). Begg's rank correlation test indicated little evidence of publication bias (*P* = 0.102 to 0.403) for summary estimates of selected APOs among women with and without syphilis (Table 2). Substantial heterogeneity was found across studies in the estimates of selected adverse outcomes for both women with syphilis (*I^2^* range: 66–95.3%; all *P*≤0.0002) and women without syphilis (*I^2^* range: 93.5–97.3%; all *P*<0.0001).

**Table 4:** Comparison for summary estimates of the proportion (%) of adverse pregnancy outcomes (APOs) among different subgroups.

Subgroup	Miscarriage		Stillbirth or fetal loss		Neonatal death	
	absolute differences	chi-square test	absolute differences	chi-square test	absolute differences	chi-square test
Women with syphilis *vs* women without syphilis	4.3%	*χ* ^2^ = 46.895, *P* = 0.000	8.8%	*χ* ^2^ = 937.960, *P* = 0.000	4.6%	*χ* ^2^ = 124.340, *P* = 0.000
Untreated women with syphilis *vs* women without syphilis	12.6%	*χ* ^2^ = 106.857, *P* = 0.000	22.7%	?^2^ = 2075.991, *P* = 0.000	14.2%	*χ* ^2^ = 415.742, *P* = 0.000
Treatment in the third trimester *vs* women without syphilis			17.6%	?^2^ = 285.499, *P* = 0.000		
High titers (≥1∶8) *vs* women without syphilis			10.9%	?^2^ = 135.901, *P* = 0.000	14.0%	*χ* ^2^ = 214.264, *P* = 0.000
Untreated women with syphilis *vs* Treated women with syphilis	11.3%	*χ* ^2^ = 42.433, *P* = 0.000	21.9%	*χ* ^2^ = 407.784, *P* = 0.000	13.0%	*χ* ^2^ = 41.721, *P* = 0.000
Treatment in the third trimester *vs* treatment in the first trimester			16.0%	*χ* ^2^ = 13.714, *P* = 0.000		
High titers (≥1∶8) *vs* Low titers (<1∶8)			11.9%	*χ* ^2^ = 66.699, *P* = 0.000	15.2%	*χ* ^2^ = 100.451, *P* = 0.000
Gestational week at treatment for women with syphilis				*χ^2^_trend_* = 29.633, *P* = 0.000		

### Subgroup analysis

The subgroup analysis was performed based on clinical characteristics: treatment or not during pregnancy, gestational week at treatment, and baseline titers of nontreponemal antibodies among women with syphilis for all APOs and selected APOs. After subgroup analysis, the heterogeneity was obviously decreased, although there was still significant heterogeneity for most of subgroups.

“untreated women with syphilis” vs “treated women with syphilis”: the pooled estimates were 76.8% (95%CI: 68.8–83.2) for all APOs, 36.0% (95%CI: 28.0–44.9) for CS, 23.2% (95%CI: 18.1–29.3) for preterm, 23.4% (95%CI: 12.8–38.6) for low birth weight, 26.4% (95%CI: 21.9–31.4) for stillbirth or early fetal loss, 14.9% (95%CI: 11.4–19.4) for miscarriage and 16.2% (95%CI: 10.1–25.1) for neonatal deaths among untreated women with syphilis, and in contrast, 24.2% (95%CI: 18.6–30.8) for all APOs, 14.0% (95%CI: 10.5–18.5) for CS, 9.9% (95%CI: 8.6–11.4) for preterm, 6.2% (95%CI: 3.9–9.8) for low birth weight, 4.5% (95%CI: 3.1–6.4) for stillbirth or early fetal loss, 3.6% (95%CI: 2.5–5.1) for miscarriage and 3.2% (95%CI: 1.1–9.1) for neonatal deaths among syphilitic women receiving treatment during pregnancy (Table 5), for the absolute differences of 52.6%, 22.0%, 13.3%, 17.2%, 21.9%, 11.3%, and 13.0% (all *P* value = 0.0000), respectively (Table 3 and Table 4). Begg's rank correlation test indicated little evidence of publication bias (*P* = 0.102 to 0.353) for summary estimates of APOs among untreated and treated women with syphilis (Table 5). Compared with women without syphilis, the untreated women with syphilis had significantly higher proportions of pregnancy loss, and the absolute differences were 63.1% for all APOs (χ^2^ = 3947.821, *P* = 0.000), 16.0% for preterm (χ^2^ = 139.350, *P* = 0.000), 18.9% for low birth weight (χ^2^ = 110.776, *P* = 0.000), 22.7% for stillbirth or early fetal loss (χ^2^ = 2075.991, *P* = 0.000), 12.6% for miscarriage (*χ* ^2^ = 106.857, *P* = 0.000) and 14.2% for neonatal deaths (*χ* ^2^ = 415.742, *P* = 0.000) (Table 3 and Table 4).

**Table 5:** Subgroup analysis based on treatment or not in pregnancy for the proportion (%) of adverse pregnancy outcomes (APOs) among syphilis-infected women.

Treatment or not in pregnancy	Reported proportion in the original studies		APOs	n	No. of included studies	Summary estimates (95%CI)#	Heterogeneity	Bias assessment
	Range	M (IQR)						
Untreated women with syphilis								
All APOs	13.9%–100.0%	82.7% (89.5%–70.4%)	1611	2651	32	76.8% (95%CI: 68.8%–83.2%)	I^2^ = 92.7%, *P*<0.0001	*P* = 0.207
Congenital syphilis	2.2%–81.8%	34.4% (68.3%–23.7%)	887	3240	33	36.0% (95%CI: 28.0%–44.9%)	I^2^ = 92.9%, *P*<0.0001	P = 0.117
Preterm birth	3.0%–62.5%	18.2% (28.7%–12.6%)	179	932	25	23.2% (95%CI: 18.1%–29.3%)	I^2^ = 6.6%, *P*<0.0001	*P* = 0.102
Low birth weight	6.8%–50.0%	29.6% (32.0%–11.3%)	63	403	8	23.4% (95%CI: 12.8%–38.6%)	I^2^ = 81.3%, *P*<0.0001	*P* = 0.104
Miscarriage	6.1%–29.4%	16.0% (20.0%–7.9%)	46	343	10	14.9% (95%CI: 11.4%–19.4%)	I^2^ = 26.4%, *P* = 0.2012	*P* = 0.172
Stillbirth or fetal loss	7.1%–66.7%	25.0% (42.1%–17.2%)	660	3001	31	26.4% (95%CI: 21.9%–31.4%)	I^2^ = 81.8%, *P*<0.0001	*P* = 0.202
Neonatal death	1.3%–60.0%	15.2% (25.9%–7.4%)	117	910	16	16.2% (95%CI: 10.1%–25.1%)	I^2^ = 81.5%, *P*<0.0001	*P* = 0.212
Treated women with syphilis*								
All APOs	2.4%–54.4%	24.5% (38.2%–15.9%)	767	3711	36	24.2% (95%CI: 18.6%–30.8%)	I^2^ = 92.5%, *P*<0.0001	*P* = 0.353
Congenital syphilis	0.7%–50.8%	13.9% (21.9%–8.2%)	621	4975	35	14.0% (95%CI: 10.5%–18.5%)	I^2^ = 91.1%, *P*<0.0001	*P* = 0.303
Preterm birth	4.7%–23.3%	14.6% (32.6%–6.0%)	180	2060	28	9.9% (95%CI: 8.6%–11.4%)	I^2^ = 43.1%, *P* = 0.0089	*P* = 0.170
Low birth weight	2.2%–15.3%	6.3% (10.7%–2.3%)	72	1457	10	6.2% (95%CI: 3.9%–9.8%)	I^2^ = 70.4%, *P* = 0.0004	*P* = 0.139
Miscarriage	2.1%–6.2%	4.0% (5.6%–3.2%)	29	862	7	3.6% (95%CI: 2.5%–5.1%)	I^2^ = 0%, *P* = 0.6896	*P* = 0.107
Stillbirth or fetal loss	1.1%–13.5%	3.4% (8.6%–2.0%)	98	2661	24	4.5% (95%CI: 3.1%–6.4%)	I^2^ = 58.5%, *P* = 0.0002	*P* = 0.219
Neonatal death	1.0%–10.3%	4.6% (8.1%–1.1%)	9	446	5	3.2% (95%CI: 1.1%–9.1%)	I^2^ = 59.0%, *P* = 0.0449	*P* = 0.111

M = Median; IQR = Inter-quartile range; APOs =  adverse pregnancy outcomes; CI = Confidence interval.

# Summary estimates and their corresponding 95% CI were calculated using either fixed-effects models or, in the presence of heterogeneity, random-effects models.

*Syphilitic women receiving at least one injection of 2.4 million units of penicillin before delivery.

“treatment in the third trimester” vs “treatment in the first trimester”: the pooled estimates of all APOs, CS, preterm, low birth weight, and stillbirth or early fetal loss were 64.4% (95%CI: 45.2–79.8), 40.6% (95%CI: 31.3–50.7), 17.6% (95%CI: 11.4–26.5), 12.4% (95%CI: 5.9–24.2), and 21.3% (95%CI: 17.2–26.0), respectively among women with syphilis receiving treatment in the third trimester (i.e.>28 weeks), and 13.3% (95%CI: 7.7–21.8), 10.4% (95%CI: 7.7–14.0), 6.8% (95%CI: 3.7–12.2), 10.0% (95%CI: 2.5–32.4), and 5.3% (95%CI: 2.2–12.1), respectively among syphilitic women who got treatment in the first trimester (i.e.≤12 weeks) (Table 6), for the absolute differences of 51.1% (*P* = 0.000), 31.1% (*P* = 0.000), 10.8% (*P* = 0.003), 2.4% (*P* = 0.889), and 16.0% (*P* = 0.000), respectively (Table 3 and Table 4). Begg's rank correlation test indicated little evidence of publication bias (*P* = 0.101 to 0.134) for summary estimates of APOs among women with syphilis according to gestational week at treatment (Table 6). Compared with non-syphilitic women (Table 3 and Table 4), the syphilis-infected women who received treatment in the third trimester also had evidently increased proportions of adverse outcomes, and the absolute differences were 50.7% for all APOs (χ^2^ = 727.296, *P* = 0.000), 10.4% for preterm (χ^2^ = 24.696, *P* = 0.000), 7.9% for low birth weight (χ^2^ = 28.440, *P* = 0.000), and 17.6% for stillbirth or early fetal loss (χ^2^ = 285.499, *P* = 0.000).

**Table 6:** Subgroup analysis based on gestational week at treatment for the proportion (%) of adverse pregnancy outcomes (APOs) among syphilis-infected women.

Gestational week at treatment	Reported proportion in the original studies		APOs	n	No. of included studies	Summary estimates (95%CI)#	Heterogeneity	Bias assessment
	Range	M (IQR)						
Treatment in the first trimester (≤12 weeks)								
All APOs	6.5%–36.0%	8.2% (20.6%–6.8%)	37	277	8	13.3% (95%CI: 7.7%–21.8%)	I^2^ = 59.8%, *P* = 0.0149	*P* = 0.114
Congenital syphilis	2.9%–20.8%	8.2% (9.4%–5.4%)	39	416	8	10.4% (95%CI: 7.7%–14.0%)	I^2^ = 32.8%, *P* = 0.1662	*P* = 0.105
Preterm birth	2.8%–12.0%	6.5% (11.0%–3.5%)	10	172	5	6.8% (95%CI: 3.7%–12.2%)	I^2^ = 0%, *P* = 0.5053	*P* = 0.110
Low birth weight			2	20	1	10.0% (95%CI: 2.5%–32.4%)		
Stillbirth or fetal loss	4.1%–8.0%	6.1%	5	99	2	5.3% (95%CI: 2.2%–12.1%)	I^2^ = 0%, *P* = 0.4445	
Treatment in the second trimester (12–28 weeks)								
All APOs	15.6%–65.1	40.0% (63.6%–22.6%)	138	447	7	37.8% (23.7%–54.3%)	I^2^ = 88.7%, *P*<0.0001	*P* = 0.102
Congenital syphilis	3.2%–44.7%	19.1% (27.8%–8.7%)	249	1359	13	17.6% (95%CI: 11.8%–25.4%)	I^2^ = 84.1%, *P*<0.0001	*P* = 0.114
Preterm birth	2.5%–25.0%	9.7% (19.6%–6.0%)	32	379	5	10.1% (95%CI: 5.2%–18.5%)	I^2^ = 65%, *P* = 0.0220	*P* = 0.101
Low birth weight	1.7%–15.0%		5	140	2	5.3% (95%CI: 0.6%–35.8%)	I^2^ = 83.6%, *P* = 0.0136	
Stillbirth or fetal loss	1.7%–7.1%	6.5%	6	179	3	4.2% (95%CI: 1.9%–9.1%)	I^2^ = 27.2%, *P* = 0.2522	*P* = 0.103
Ttreatment in the third trimester (>28 weeks)								
All APOs	12.0%–100.0%	68.2% (94.4%–34.5%)	292	540	11	64.4% (95%CI: 45.2%–79.8%)	I^2^ = 91.6%, *P*<0.0001	*P* = 0.116
Congenital syphilis	18.2%–83.3%	45.0% (60.0%–26.5%)	428	1454	15	40.6% (95%CI: 31.3%–50.7%)	I^2^ = 87%, *P*<0.0001	*P* = 0.131
Preterm birth	5.3%–35.0%	20.6% (26.9%–12.9%)	65	447	7	17.6% (95%CI: 11.4%–26.5%)	I^2^ = 69.2%, *P* = 0.0035	*P* = 0.134
Low birth weight	3.4%–26.9%	12.8% (23.9%–5.2%)	26	236	4	12.4% (95%CI: 5.9%–24.2%)	I^2^ = 66.1%, *P* = 0.0315	*P* = 0.101
Stillbirth or fetal loss	17.7%–40.0%	22.9% (27.7%–18.9%)	71	336	6	21.3% (95%CI: 17.2%–26.0%)	I^2^ = 0%, *P* = 0.8177	*P* = 0.107

M = Median; IQR = Inter-quartile range; APOs =  adverse pregnancy outcomes; CI = Confidence interval.

# Summary estimates and their corresponding 95% CI were calculated using either fixed-effects models or, in the presence of heterogeneity, random-effects models.

“high titers” vs “low titers”: the pooled estimates were 42.8% (95%CI: 26.2–61.2) for all APOs, 25.8% (95%CI: 15.4–40.1) for CS, 15.1% (95%CI: 5.2–36.9) for preterm, 9.4% (95%CI: 2.7–27.5) for low birth weight, 14.6% (95%CI: 6.5–29.7) for stillbirth or early fetal loss and 16.0% (95%CI: 12.0–21.1) for neonatal deaths among syphilitic women with high titers, and 11.0% (95%CI: 6.3–18.5) for all APOs, 4.2% (95%CI: 1.9–9.1) for CS, 2.9% (95%CI: 0.8–10.2) for preterm, 3.9% (95%CI: 2.7–5.5) for low birth weight, 2.7% (95%CI: 0.4–15.3) for stillbirth or early fetal loss and 0.8% (95%CI: 0.1–10.2) for neonatal deaths among syphilitic women with low titers (Table 7), for the absolute differences of 31.8% (χ^2^ = 174.840, *P* = 0.000), 21.6% (χ^2^ = 283.664, *P* = 0.000), 12.2% (*χ* ^2^ = 55.631, *P* = 0.000), 5.5% (*χ* ^2^ = 13.853, *P* = 0.000), 11.9% (*χ* ^2^ = 66.699, *P* = 0.000), and 15.2% (*χ* ^2^ = 100.451, *P* = 0.000), respectively (Table 3 and Table 4). Begg's rank correlation test indicated little evidence of publication bias (*P* = 0.102 to 0.210) for summary estimates of APOs among women with syphilis according to baseline titers of nontreponemal antibodies (Table 7).Similarly, when compared with women without syphilis, the syphilitic women with high titers also had significantly higher proportions of all APOs (all *P* value = 0.0000), preterm, low birth weight, stillbirth or early fetal loss as well as neonatal deaths, and the absolute differences were 29%, 8%, 4%, 13%, and 14%, respectively (Table 3 and Table 4).

**Table 7:** Subgroup analysis based on baseline titers of nontreponemal antibodies for the proportion (%) of adverse pregnancy outcomes (APOs) among syphilis-infected women.

Maternal baseline titers of nontreponemal antibodies	Reported proportion in the original studies		APOs	n	No. of included studies	Summary estimates (95%CI)#	Heterogeneity	Bias assessment
	Range	M (IQR)						
Low titers (<1∶8)								
All APOs	3.7%–24.1%	9.3% (21.9%–4.9%)	114	1215	6	11.0% (95%CI: 6.3%–18.5%)	I^2^ = 87%, *P*<0.0001	*P* = 0.131
Congenital syphilis	0.2%–21.9%	4.1% (14.0%–1.3%)	251	3085	8	4.2% (95%CI: 1.9%–9.1%)	I^2^ = 94.4%, *P*<0.0001	*P* = 0.210
Preterm birth	0.5%–9.3%	3.6% (8.3%–0.9%)	32	998	3	2.9% (95%CI: 0.8%–10.2%)	I^2^ = 88%, *P*<0.0001	*P* = 0.130
Low birth weight	3.4%–5.2%	3.7%	31	813	3	3.9% (95%CI: 2.7%–5.5%)	I^2^ = 0%, *P* = 0.5164	*P* = 0.127
Stillbirth or fetal loss	0.7%–7.8%	3.7%	20	813	3	2.7% (95%CI: 0.4%–15.3%)	I^2^ = 89.9%, *P*<0.0001	*P* = 0.102
Neonatal death	0.2%–2.6%	1.4%	4	708	2	0.8% (95%CI: 0.1%–10.2%)	I^2^ = 82.5%, *P* = 0.0167	
High titers (≥1∶8)								
All APOs	15.2%–73.7%	49.3% (63.9%–21.9%)	182	510	6	42.8% (95%CI: 26.2%–61.2%)	I^2^ = 92.2%, *P*<0.0001	*P* = 0.111
Congenital syphilis	2.2%–72.2%	25.2% (40.7%–15.8%)	325	1161	8	25.8% (95%CI: 15.4%–40.1%)	I^2^ = 94.4%, *P*<0.0001	*P* = 0.152
Preterm birth	2.2%–37.5%	20.0% (34.4%–5.5%)	51	359	3	15.1% (95%CI: 5.2%–36.9%)	I^2^ = 91.8%, *P*<0.0001	*P* = 0.112
Low birth weight	4.5%–24.7%	6.3%	32	347	3	9.4% (95%CI: 2.7%–27.5%)	I^2^ = 90.9%, *P*<0.0001	*P* = 0.105
Stillbirth or fetal loss	6.2%–34.2%	12.8% (29.2%–7.6%)	57	383	3	14.6% (95%CI: 6.5%–29.7%)	I^2^ = 88%, *P*<0.0001	*P* = 0.105
Neonatal death	15.2%–18.1%	16.7%	40	250	2	16.0% (95%CI: 12.0%–21.1%)	I^2^ = 0%, *P* = 0.5733	

M = Median; IQR = Inter-quartile range; APOs =  adverse pregnancy outcomes; CI = Confidence interval.

# Summary estimates and their corresponding 95% CI were calculated using either fixed-effects models or, in the presence of heterogeneity, random-effects models.

## Discussion

In the present study, we quantified the proportion of all APOs and specific APOs among syphilis-infected women and non-syphilitic women using data from fifty-four studies that met eligibility criteria for inclusion in our systematic review and meta-analysis and involved 11398 women with syphilis and 43342 women without syphilis. In the context of WHO's global initiative for the elimination of CS and National Program for Prevention and Control of Syphilis in China (2010–2020), this study could supply helpful information to both clinical doctors and infected mothers, and help to assess progress in elimination of MTCT of syphilis and to guide policy and advocacy efforts. To our knowledge, this is the first time that the epidemic of APOs among women with syphilis was exhaustively reviewed based on clinical features by meta-analysis.

Findings from present study further confirmed the ancient tune that MTCT of syphilis undoubtedly brings about a heavy burden to society. Notwithstanding being easily detectable and treatable in pregnancy, presently, syphilis remains an important cause of birth loss [1], [20], [24]. On average, our review showed that APOs accounted for significantly higher proportions among the offspring of syphilis-infected mothers than among the offspring of mothers without syphilis, especially among syphilis-infected women who didn't receive treatment during pregnancy, or who did not receive treatment until the third trimester, or who had high baseline titers.

Previous studies have confirmed that lack of treatment or postponement of gestational week for first treatment and high baseline titers were independent risk factors of APOs among syphilitic mothers [1], [25]. Our study indicates that, unless testing and treatment of syphilis in pregnancy are universally available, over half of pregnancies in women with syphilis will result in an adverse outcome. In general, our estimates are consistent with previously published data. In order to block MTCT of syphilis and support the global initiative for elimination of CS, WHO has developed 2008 worldwide estimates of maternal syphilis and associated APOs, which indicated that, globally, 520,905 adverse outcomes were estimated to be caused by maternal syphilis, including approximately 212,327 stillbirths or early fetal deaths, 91,764 neonatal deaths, 65,267 preterm or low birth weight infants, and 151,547 infected newborns [9]. Furthermore, WHO also revealed that approximately 66% of adverse outcomes occurred in antenatal care (ANC) attendees who were not tested or were not treated for syphilis, and in 2008, based on the middle case scenario, clinical services likely averted 26% of all APOs [9]. The latest meta-analysis by Gomez et al. [8] showed that approximately 52% of pregnancies in mothers with untreated or inadequately treated syphilis result in some APOs, with estimated proportions: early fetal loss or stillbirth (21%), neonatal death (9%), low birth weight or premature birth (6%), and infection in a live-born infant (15%). Berman [26] indicated that if left untreated, maternal syphilis infection will, in up to 80% of pregnancies, lead to severely APOs, including stillbirth, premature birth, neonatal death, or congenital infection in the newborn. Qin JB et al. [1] confirmed that for mothers who did not receive complete treatment during pregnancy, 18.5% delivered an infant with CS and 48.1% resulted in APOs. It has been estimated that the numbers of fetal/neonatal deaths in Africa each year from untreated maternal syphilis could rival those from HIV infections [27].

Both previous studies and present review indicated that improving quality of ANC is a key point to eliminate MTCT of syphilis and reduce the risk of APOs among women with syphilis. This highlights the importance of absent or insufficient ANC as an independent risk factor for pregnancy loss among syphilis-infected mothers. ANC is not only the best opportunity to treat maternal syphilis, but it is also important for the control of a woman who had received documented adequate treatment for syphilis before pregnancy, and is necessary for the interpretation of a positive serologic test at delivery [28]. Screening and treatment of syphilis during pregnancy is considered to be simple, cheap, and highly cost-effective in prevention of MTCT of syphilis. For these reasons, screening pregnant women during their first ANC is recommended by the WHO [29]. However, presently, approximately one-fifth (20%) of all pregnant women with syphilis did not attend ANC [9]. It is also recognized that the effectiveness of screening and treatment is lower in the third trimester than in the first and second trimesters [30]. Given that screening and treatment for preventing MTCT of syphilis is not 100% effective, primary prevention of syphilis in pregnant women is also an important strategy that needs to be addressed to truly eliminate MTCT of syphilis. Although substantial progress has been made in the utilization of ANC (in 2009 WHO estimated that approximately 81% of all pregnant women had attended at least one ANC visit [9]), MTCT of syphilis occurred for a variety of reasons: many of these visits were too late to avert an adverse outcome, clinics may not have offered testing, testing may not have been affordable, women may not have followed up or received their test results, treatment may not have been available, or treated women may have been reinfected by untreated sexual partners [31].

Our estimates are subject to certain limitations that should be considered when interpreting the results. Firstly, all studies included in our review had an observational design, and most belonged to a retrospective cases analysis. Owing to the inherent differences between experimental and observational study designs and to biases commonly seen in observational data, appropriate caution should be taken in interpreting our results. Nevertheless, an advantage of this review is that we included a comparison group to assess APOs among mothers without syphilis. This gave us the opportunity to estimate the excess adverse outcomes in the presence of maternal syphilis and give a broad idea of the risk of some of the pregnancy loss in syphilis-infected women. Secondly, there was also unacceptable heterogeneity in estimates across studies. We tried to find the sources of heterogeneity by subgroup analysis. After subgroup analysis, the heterogeneity was obviously decreased. However, our estimates have to be viewed with caution because of heterogeneity. Thirdly, because variations in the definition of APOs exist across countries and cultures, it is extremely difficult to define uniform standards. The early literatures did not always define birth outcomes and in such cases we relied on the outcome terminology in the original papers. So the misclassification of APOs may influence the results. Last but not least, our relatively strict inclusion criteria might have introduced selection bias. In present analysis, we only included studies published in Chinese or English language. So the additional research in other populations is warranted to generalize the findings. The limitations of these estimates highlight the urgent need for improved data through stronger national surveillance and monitoring systems.

In summary, present study indicates that syphilis continues to be an important cause of substantial numbers of perinatal deaths and disabilities that could be prevented by early testing and treatment, and also reminds policy-makers charged with resource allocation that the elimination of MTCT of syphilis is a public health priority. Most adverse outcomes occurred among women who were not treated for syphilis or who receiving treatment only in the late trimester or who had high baseline titers. High quality of ANC highlighting early testing and treatment is the only effective means to block MTCT of syphilis. Health education for pregnant women should continue to reinforce the message that untreated maternal syphilis is a danger to the unborn infant, that syphilis can be diagnosed and treated, and that women should attend an antenatal that can perform syphilis screening as soon as they suspect that they are pregnant. Systematic attention to testing, treatment, education, and contact tracing in pregnancy and subsequent late trimester retesting of women at high risk will lower pregnancy loss.

## Supporting Information

Checklist S1 **PRISMA checklist.**
(DOC)

Diagram S1 **PRISMA Flow Diagram.**
(DOC)
