# Supplementary material for: Reported Estimates of Adverse Pregnancy Outcomes among Women with and without Syphilis: A Systematic Review and Meta-Analysis
Source: PLoS One. 2014 Jul 15;9(7):e102203. doi: 10.1371/journal.pone.0102203 (PMC4099012; doi:10.1371/journal.pone.0102203)
Supplement: Checklist S1 — PRISMA checklist. (DOC) [file pone.0102203.s001.doc]

| **Section/topic** | **#** | **Checklist item** | **Reported on page #** |
| --- | --- | --- | --- |
| **TITLE** | | |  |
| Title | 1 | Identify the report as a systematic review, meta-analysis, or both. | Reported estimates of adverse pregnancy outcomes among women with and without syphilis: a systematic review and meta-analysis |
| **ABSTRACT** | | |  |
| Structured summary | 2 | Provide a structured summary including, as applicable: background; objectives; data sources; study eligibility criteria, participants, and interventions; study appraisal and synthesis methods; results; limitations; conclusions and implications of key findings; systematic review registration number. | Background: To estimate probability of adverse pregnancy outcomes(APOs) among women with and without syphilis through a systematic review of published literatures.Methodology/Principal Findings: Chinese and English literatures were searched for studies assessing pregnancy outcomes in the presence of maternal syphilis through August 2013. The prevalence estimates were summarized and analyzed by meta-analysis. Fifty-four literatures involving 11398 syphilitic women and 43342 non-syphilitic women were included from 4187 records initially found. Among untreated mothers with syphilis, pooled estimates were 76.8% for all APOs, 36.0% for congenital syphilis, 23.2% for preterm, 23.4% for low birth weight, 26.4% for stillbirth or fetal loss, 14.9% for miscarriage and 16.2% for neonatal deaths. Among syphilitic mother receiving treatment only in the late trimester(>28 weeks), pooled estimates were 64.4% for APOs, 40.6% for congenital syphilis, 17.6% for preterm, 12.4% for low birth weight, and 21.3% for stillbirth or fetal loss. Among syphilitic mothers with high titers(≥1:8), pooled estimates were 42.8% for all APOs, 25.8% for congenital syphilis, 15.1% for preterm, 9.4% for low birth weight, 14.6% for stillbirth or fetal loss and 16.0% for neonatal deaths. Among non-syphilitic mothers, the pooled estimates were 13.7% for all APOs, 7.2% for preterm birth, 4.5% for low birth weight, 3.7% for stillbirth or fetal loss, 2.3% for miscarriage and 2.0% for neonatal death. Begg’s rank correlation test indicated little evidence of publication bias(P>0.10). Substantial heterogeneity was found across studies in the estimates of all adverse outcomes for both women with syphilis (I2=93.9%; P<0.0001) and women without syphilis (I2=94.8%; P <0.0001).Conclusions/Significance: Syphilis continues to be an important cause of substantial perinatal morbidity and mortality, which reminds that policy-makers charged with resource allocation that the elimination of mother-to-child transmission of syphilis is a public health priority. |
| **INTRODUCTION** | | |  |
| Rationale | 3 | Describe the rationale for the review in the context of what is already known. | Currently, although some estimates for burden of syphilis in pregnancy and associated APOs were available, the global incidence of adverse birth outcomes among syphilitic women remains enough unclear. Over the years, some researchers have been trying to estimate the incidence of APOs resulting from maternal syphilis. For example, the latest meta-analysis by Gomez et al.[8] that is currently the only systematic review assessing this question indicated that approximately 52% of pregnancies in mothers with untreated or inadequately treated syphilis result in some APOs, with estimated proportions: early fetal loss or stillbirth (21%), neonatal death (9%), low birth weight or premature birth (6%), and infection in a live-born infant (15%), and among untreated pregnant women with syphilis, fetal loss and stillbirth were 21% more frequent, neonatal deaths were 9.3% more frequent and prematurity or low birth weight were 5.8% more frequent than among women without syphilis. However, this review didn’t assess APOs under the background of different baseline titers and treatment time for maternal syphilis, and didn’t include Chinese literatures. In China, there are large study reports to assess adverse outcomes among women with syphilis, while China’s literatures are mainly local or single medical institution reports and only include the small sample size of study population, which causes that the findings are not comprehensive and meaningful representation of poor. Overall, most of previous estimates of APOs in women with syphilis have been based on point estimates from single studies. |
| Objectives | 4 | Provide an explicit statement of questions being addressed with reference to participants, interventions, comparisons, outcomes, and study design (PICOS). | In order to support the global initiative for elimination of MTCT of syphilis, we conducted a systematic review and meta-analysis with the following objectives: (1) to estimate the APOs among syphilitic women according to baseline titers, treatment or not during pregnancy and gestational age at treatment; (2) to estimate APOs among women without syphilis; and (3) to provide scientific evidence for the prevention of pregnancy loss attributable to MTCT of syphilis. |
| **METHODS** | | |  |
| Protocol and registration | 5 | Indicate if a review protocol exists, if and where it can be accessed (e.g., Web address), and, if available, provide registration information including registration number. | no |
| Eligibility criteria | 6 | Specify study characteristics (e.g., PICOS, length of follow-up) and report characteristics (e.g., years considered, language, publication status) used as criteria for eligibility, giving rationale. | For those studies that not only reported the incidence of birth outcomes among syphilis-infected women, but also reported the incidence among non-syphilitic women, we will meanwhile estimate the range of possible birth outcomes among non-syphilitic women. Our APOs of interest were CS, preterm, low birth weight, stillbirth or early fetal loss, miscarriage and neonatal death. |
| Information sources | 7 | Describe all information sources (e.g., databases with dates of coverage, contact with study authors to identify additional studies) in the search and date last searched. | PubMed, Cochrane Libraries, China Biology Medicine disc (CBMdisc), Chinese Scientific Journals Fulltext Database (CQVIP), China National Knowledge Infrastructure (CNKI) and Wanfang Data were searched through August 2013 with no restrictions to identify published peer-reviewed research articles assessing pregnancy outcomes in the presence of maternal syphilis by the following search terms: syphilis, pregnancy, adverse birth or pregnancy outcomes, congenital syphilis, preterm, low birth weight, stillbirth, fetal loss or death, abortion or miscarriage, neonatal death, and perinatal death or morbidity or mortality. We also performed a manual search on the reference lists of published articles. The grey literature and conference abstracts were not searched. |
| Search | 8 | Present full electronic search strategy for at least one database, including any limits used, such that it could be repeated. | PubMed, Cochrane Libraries, China Biology Medicine disc (CBMdisc), Chinese Scientific Journals Fulltext Database (CQVIP), China National Knowledge Infrastructure (CNKI) and Wanfang Data were searched through August 2013 with no restrictions to identify published peer-reviewed research articles assessing pregnancy outcomes in the presence of maternal syphilis by the following search terms: syphilis, pregnancy, adverse birth or pregnancy outcomes, congenital syphilis, preterm, low birth weight, stillbirth, fetal loss or death, abortion or miscarriage, neonatal death, and perinatal death or morbidity or mortality. We also performed a manual search on the reference lists of published articles. The grey literature and conference abstracts were not searched. |
| Study selection | 9 | State the process for selecting studies (i.e., screening, eligibility, included in systematic review, and, if applicable, included in the meta-analysis). | Studies were considered eligible for inclusion in this systematic review if they met the following criteria: (1) studies published in Chinese or English language; (2) studies described pregnancy outcomes among women presumed to have syphilis(i.e. women who were seroreactive for T. pallidum infection, irrespective of the test used); (3) study populations excluded HIV-positive women; (4) sample size for cases was more than 30 syphilitic patients; and (5) the incidences of APOs were reported(or data to calculate them). We excluded review papers, non peer–reviewed local/government reports, conference abstract and presentation in this study. If the same study data were published in both English and Chinese sources, the articles published in Chinese language were excluded from the review. We considered a broad range of study designs, including clinical tri­als, observational studies, and case series. We also assessed potential studies to ensure that there was no duplication of case series. |
| Data collection process | 10 | Describe method of data extraction from reports (e.g., piloted forms, independently, in duplicate) and any processes for obtaining and confirming data from investigators. | Two independent reviewers (JBQ and HLF) assessed eligibility criteria and extracted data, and any disagreements were resolved by discussion. We extracted the following information from all eligible studies: first author and published year; geographical location; study design; study period; syphilis prevalence among mothers; subgroup variables (infection status of syphilis, treatment or not for maternal syphilis, gestational age at treatment, and baseline titers of nontreponemal antibodies); sample size for cases and controls; reported adverse outcomes. Because variations in the definition of APOs exist across countries and cultures, it is extremely difficult to define uniform standards. The early literatures did not always define birth outcomes and in such cases we relied on the outcome terminology in the original papers. |
| Data items | 11 | List and define all variables for which data were sought (e.g., PICOS, funding sources) and any assumptions and simplifications made. | We extracted the following information from all eligible studies: first author and published year; geographical location; study design; study period; syphilis prevalence among mothers; subgroup variables (infection status of syphilis, treatment or not for maternal syphilis, gestational age at treatment, and baseline titers of nontreponemal antibodies); sample size for cases and controls; reported adverse outcomes. Because variations in the definition of APOs exist across countries and cultures, it is extremely difficult to define uniform standards. The early literatures did not always define birth outcomes and in such cases we relied on the outcome terminology in the original papers. |
| Risk of bias in individual studies | 12 | Describe methods used for assessing risk of bias of individual studies (including specification of whether this was done at the study or outcome level), and how this information is to be used in any data synthesis. | Begg’s rank correlation test was used to assess publication bias(p<0.10 represents statistical significance). |
| Summary measures | 13 | State the principal summary measures (e.g., risk ratio, difference in means). | We calculated the combined incidence and the corresponding 95% confidence intervals (CI) for all APOs in women with syphilis and women without syphilis. We then also calculated the summary incidence and the corresponding 95%CI for the following selected pregnancy loss. |
| Synthesis of results | 14 | Describe the methods of handling data and combining results of studies, if done, including measures of consistency (e.g., I^2^) for each meta-analysis. | The combined incidence and the corresponding 95 % CI were calculated using either fixed-effects models or, in the presence of heterogeneity, random-effects models. Heterogeneity tests were performed using the Cochran Q-test (p<0.10 represents statistically significant heterogeneity) and I2 statistic. Begg’s rank correlation test was used to assess publication bias(p<0.10 represents statistical significance). The chi-square test was used to analyze the difference between subgroups(p<0.05 represents statistical significance). The comparison between subgroups was performed using SAS version 9.1, and other data were prepared and analyzed using R software version 3.0. |

Page 1 of 2

| **Section/topic** | **#** | **Checklist item** | **Reported on page #** |
| --- | --- | --- | --- |
| Risk of bias across studies | 15 | Specify any assessment of risk of bias that may affect the cumulative evidence (e.g., publication bias, selective reporting within studies). | Begg’s rank correlation test was used to assess publication bias(p<0.10 represents statistical significance). |
| Additional analyses | 16 | Describe methods of additional analyses (e.g., sensitivity or subgroup analyses, meta-regression), if done, indicating which were pre-specified. | The subgroup analysis for all APOs and specific APOs was performed based on whether women were infected with syphilis, whether syphilitic women were treated during pregnancy(i.e. syphilitic women receiving at least one injection of 2.4 million units of penicillin before delivery), gestational week at treatment(i.e. <12 or 12 to 28 or ≥28 weeks), and maternal baseline titers(i.e. ≥1:8 or <1:8) to explore the sources of heterogeneity. |
| **RESULTS** | | |  |
| Study selection | 17 | Give numbers of studies screened, assessed for eligibility, and included in the review, with reasons for exclusions at each stage, ideally with a flow diagram. | Our initial search criteria identified 4149 articles from six electronic databases and 38 additional articles were identified through reference lists from identified articles. Of these, the majority were excluded after the first screening based on abstracts or titles, mainly because they were review papers, and unrelated to the topics or duplicated titles from different databases(Figure 1). Finally, fifty-four studies were considered eligible in qualitative synthesis(Table 1). |
| Study characteristics | 18 | For each study, present characteristics for which data were extracted (e.g., study size, PICOS, follow-up period) and provide the citations. | Finally, fifty-four studies were considered eligible in qualitative synthesis(Table 1). |
| Risk of bias within studies | 19 | Present data on risk of bias of each study and, if available, any outcome level assessment (see item 12). | Begg’s rank correlation test was used to assess publication bias(p<0.10 represents statistical significance). The subgroup analysis for all APOs and specific APOs was performed based on whether women were infected with syphilis, whether syphilitic women were treated during pregnancy(i.e. syphilitic women receiving at least one injection of 2.4 million units of penicillin before delivery), gestational week at treatment(i.e. <12 or 12 to 28 or ≥28 weeks), and maternal baseline titers(i.e. ≥1:8 or <1:8) to explore the sources of heterogeneity. |
| Results of individual studies | 20 | For all outcomes considered (benefits or harms), present, for each study: (a) simple summary data for each intervention group (b) effect estimates and confidence intervals, ideally with a forest plot. | All pools estimates and 95% confidence intervals for adverse pregnancy outcomes among women with and without syphilis were summarized in table 2 to table 7. |
| Synthesis of results | 21 | Present results of each meta-analysis done, including confidence intervals and measures of consistency. | The meta-analysis for prevalence of adverse pregnancy outcomes among women with and without syphilis have been performed in the present study, and all results were summarized in  table 2 to table 7 |
| Risk of bias across studies | 22 | Present results of any assessment of risk of bias across studies (see Item 15). | Begg’s rank correlation test indicated little evidence of publication bias(all *P*>0.10). |
| Additional analysis | 23 | Give results of additional analyses, if done (e.g., sensitivity or subgroup analyses, meta-regression [see Item 16]). | We did not perform sensitivity analysis in the present study. After subgroup analysis, we found that most adverse pregnancy outcomes occurred among women who were not treated for syphilis or who receiving treatment only in the late trimester or who had high baseline titers. Such as, among untreated mothers with syphilis, pooled estimates were 76.8% for all APOs, 36.0% for congenital syphilis, 23.2% for preterm, 23.4% for low birth weight, 26.4% for stillbirth or fetal loss, 14.9% for miscarriage and 16.2% for neonatal deaths. Among syphilitic mother receiving treatment only in the late trimester(>28 weeks), pooled estimates were 64.4% for APOs, 40.6% for congenital syphilis, 17.6% for preterm, 12.4% for low birth weight, and 21.3% for stillbirth or fetal loss. Among syphilitic mothers with high titers(≥1:8), pooled estimates were 42.8% for all APOs, 25.8% for congenital syphilis, 15.1% for preterm, 9.4% for low birth weight, 14.6% for stillbirth or fetal loss and 16.0% for neonatal deaths. Among non-syphilitic mothers, the pooled estimates were 13.7% for all APOs, 7.2% for preterm birth, 4.5% for low birth weight, 3.7% for stillbirth or fetal loss, 2.3% for miscarriage and 2.0% for neonatal death. |
| **DISCUSSION** | | |  |
| Summary of evidence | 24 | Summarize the main findings including the strength of evidence for each main outcome; consider their relevance to key groups (e.g., healthcare providers, users, and policy makers). | In the present study, we quantified the propor­tion of all APOs and specific APOs among syphilis-infected women and non-syphilitic women using data from fifty-four studies that met eligibility criteria for inclusion in our systematic review and meta-analysis and involved 11398 women with syphilis and 43342 women without syphilis. In the context of WHO’s global initiative for the elimination of CS and National Program for Prevention and Control of Syphilis in China(2010–2020), this study could supply helpful information to both clinical doctors and infected mothers, and help to assess progress in elimination of MTCT of syphilis and to guide policy and advocacy efforts. To our knowledge, this is the first time that the epidemic of APOs among women with syphilis was exhaustively reviewed based on clinical features by meta-analysis. Among untreated mothers with syphilis, pooled estimates were 76.8%(95%CI:68.8-83.2) for all APOs, 36.0%(95%CI:28.0-44.9) for congenital syphilis, 23.2%(95%CI:18.1-29.3) for preterm, 23.4%(95%CI:12.8-38.6) for low birth weight, 26.4%(95%CI:21.9-31.4) for stillbirth or fetal loss, 14.9%(95%CI:11.4-19.4) for miscarriage and 16.2%(95%CI:10.1-25.1) for neonatal deaths. Among syphilitic mother receiving treatment only in the late trimester(>28 weeks), pooled estimates were 64.4%(95%CI: 45.2-79.8) for APOs, 40.6%(95%CI: 31.3-50.7) for congenital syphilis, 17.6%(95%CI: 11.4-26.5) for preterm, 12.4%(95%CI: 5.9-24.2) for low birth weight, and 21.3%(95%CI: 17.2-26.0) for stillbirth or fetal loss. Among syphilitic mothers with high titers(≥1:8), pooled estimates were 42.8%(95%CI: 26.2-61.2) for all APOs, 25.8%(95%CI: 15.4-40.1) for congenital syphilis, 15.1%(95%CI: 5.2-36.9) for preterm, 9.4%(95%CI: 2.7-27.5) for low birth weight, 14.6%(95%CI: 6.5-29.7) for stillbirth or fetal loss and 16.0%(95%CI: 12.0-21.1) for neonatal deaths. Among non-syphilitic mothers, the pooled estimates were 13.7% for all APOs, 7.2%(95%CI: 5.6-9.3) for preterm birth, 4.5%(95%CI:2.0-10.0) for low birth weight, 3.7%(95%CI: 2.6-5.1) for stillbirth or fetal loss, 2.3%(95%CI: 1.8-3.0) for miscarriage and 2.0%(95%CI: 1.2-3.3) for neonatal death. Begg’s rank correlation test indicated little evidence of publication bias(P>0.10). Substantial heterogeneity was found across studies in the estimates of all adverse outcomes for both women with syphilis (I2=93.9%; P<0.0001) and women without syphilis (I2=94.8%; P <0.0001). |
| Limitations | 25 | Discuss limitations at study and outcome level (e.g., risk of bias), and at review-level (e.g., incomplete retrieval of identified research, reporting bias). | Firstly, all studies included in our review had an observational design, and most belonged to a retrospective cases analysis. Owing to the inherent differences between experimental and observa­tional study designs and to biases com­monly seen in observational data, appropriate caution should be taken in interpreting our results. Nevertheless, an advantage of this review is that we included a comparison group to assess APOs among mothers without syphilis. This gave us the op­portunity to estimate the excess adverse outcomes in the presence of maternal syphilis and give a broad idea of the risk of some of the pregnancy loss in syphilis-infected women. Secondly, there was also unacceptable heterogeneity in estimates across studies. We tried to find the sources of heterogeneity by subgroup analysis. After subgroup analysis, the heterogeneity was obviously decreased. However, our estimates have to be viewed with caution because of heterogeneity. Thirdly, because variations in the definition of APOs exist across countries and cultures, it is extremely difficult to define uniform standards. The early literatures did not always define birth outcomes and in such cases we relied on the outcome terminology in the original papers. So the misclassification of APOs may influence the results. Last but not least, our relatively strict inclusion criteria might have introduced selection bias. In present analysis, we only included studies published in Chinese or English language. So the additional research in other populations is warranted to generalize the findings. The limitations of these estimates highlight the urgent need for improved data through stronger national surveillance and monitoring systems. |
| Conclusions | 26 | Provide a general interpretation of the results in the context of other evidence, and implications for future research. | In summary, present study indicates that syphilis continues to be an important cause of substantial numbers of perinatal deaths and disabilities that could be prevented by early testing and treatment, and also reminds policy-makers charged with resource allocation that the elimination of MTCT of syphilis is a public health priority. Most adverse outcomes occurred among women who were not treated for syphilis or who receiving treatment only in the late trimester or who had high baseline titers. High quality of ANC highlighting early testing and treatment is the only effective means to block MTCT of syphilis. |
| **FUNDING** | | |  |
| Funding | 27 | Describe sources of funding for the systematic review and other support (e.g., supply of data); role of funders for the systematic review. | J-B Qin was supported by the Fundamental Research Funds for Central South University (2012zzts029) and the Hunan Province Innovation Projects(CX2012B076) of China. This work was also supported by the Program of Prevention of mother-to-children transmission of Syphilis in Shenzhen, China. The funders had no role in study design, data collection and analysis, decision to publish, or preparation of the manuscript. |

*From:*  Moher D, Liberati A, Tetzlaff J, Altman DG, The PRISMA Group (2009). Preferred Reporting Items for Systematic Reviews and Meta-Analyses: The PRISMA Statement. PLoS Med 6(6): e1000097. doi:10.1371/journal.pmed1000097

For more information, visit: **www.prisma-statement.org**.

Page 2 of 2
