# Supplementary material for: Reported Estimates of Adverse Pregnancy Outcomes among Women with and without Syphilis: A Systematic Review and Meta-Analysis
Source: PLoS One. 2014 Jul 15;9(7):e102203. doi: 10.1371/journal.pone.0102203 (PMC4099012; doi:10.1371/journal.pone.0102203)
Supplement: Diagram S1 — PRISMA Flow Diagram. (DOC) [file pone.0102203.s002.doc]

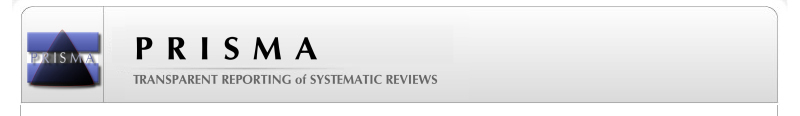
**PRISMA 2009 Flow Diagram**

**Screening**

**Included**

**Eligibility**

**Identification**

Records identified through database searches (Pubmed, Cochrane libraries, CBMdisc, CQVIP, CNKI and Wanfang Data )

(n=4149)

Additional records identified through other sources
(n = 38)

Records after duplicates removed
(n = 2709)

Records screened
(n = 2709)

Records excluded after screening of abstracts
(n =2507)

Unrelated to the topics (n=2261)

Review papers (n=246)

Full-text articles assessed for eligibility
(n =202)

Full-text articles excluded, with reasons(n=148)

HIV-positive populations: n=103

Number of patients <30: n=23

Incomplete data: n=18

Duplication: n=4

Studies included in qualitative synthesis
(n =54)

English: 12; Chinese: 42

Studies included in quantitative synthesis (meta-analysis)
(n=54)
